# Effect of Signal-Strength Filtering on 3D Convolutional Neural Network–Based Visual Field Estimation From Macular OCT

**DOI:** 10.1167/tvst.15.7.17

**Published:** 2026-07-13

**Authors:** Makoto Koyama, Hidenori Takahashi, Satoru Inoda, Chihiro Mayama, Yuta Ueno, Yoshikazu Ito, Yuta Nariya, Yuji Inoue, Kenji Kashiwagi, Tetsuro Oshika, Masaki Tanito

**Affiliations:** 1 Minamikoyasu Eye Clinic, Kimitsu, Japan; 2 Center for Cyber Medicine Research, Institute of Medicine, University of Tsukuba, Tsukuba, Japan; 3 Department of Ophthalmology, Jichi Medical University, Shimotsuke, Japan; 4 Department of Ophthalmology, Japan Community Health Care Organization Tokyo Shinjuku Medical Center, Tokyo, Japan; 5 Department of Ophthalmology, Faculty of Medicine, University of Tsukuba, Tsukuba, Japan; 6 Department of Ophthalmology, University of Tokyo Hospital, Tokyo, Japan; 7 Kameda Medical Center, Kamogawa, Chiba, Japan; 8 Department of Ophthalmology, Teikyo University, Itabashi-ku, Tokyo, Japan; 9 Department of Ophthalmology, University of Yamanashi Faculty of Medicine, Yamanashi, Japan; 10 Department of Ophthalmology, Shimane University Faculty of Medicine, Izumo, Japan

**Keywords:** visual field, optical coherence tomography, signal strength index, artificial intelligence, deep learning

## Abstract

**Purpose:**

To determine whether excluding low-signal-strength optical coherence tomography (OCT) scans from training improves deep learning–based visual field (VF) estimation from macular OCT volumes.

**Methods:**

We retrospectively analyzed 79,803 paired OCT and VF examinations from 8511 patients at five institutions. A three-dimensional convolutional neural network (3DCNN) estimated 24-2 and 10-2 pointwise sensitivities and mean deviation (MD) from NIDEK OCT volumes. Using patient-wise 10-fold cross-validation, we compared training on all scans with filtering at signal strength indices (SSIs) ≥ 7; training sets differed in size and SSI composition, and both models were tested on identical held-out samples stratified by SSI. Outcomes were pointwise mean absolute error (MAE) and absolute MD error.

**Results:**

Mean SSI was 8.03 ± 1.65; 85.5% of scans had SSI ≥ 7. In the SSI < 7 subgroup, the all-SSI-trained model yielded lower errors for all endpoints, with reductions of 0.439 dB (24-2 MAE), 0.473 dB (10-2 MAE), 0.417 dB (24-2 MD error), and 0.453 dB (10-2 MD error; all adjusted *P* < 0.001). In the SSI ≥ 7 subgroup, no significant differences were observed. Error increased as SSI decreased, without a clear threshold at SSI = 7.

**Conclusions:**

In this EfficientNet 3D-based segmentation-free 3DCNN framework, using OCT scans across the full SSI range was associated with lower error in low-SSI held-out scans, with no significant penalty in higher SSI held-out scans in the primary comparison within this dataset.

**Translational Relevance:**

Including lower-signal-strength NIDEK macular OCT scans in development may improve robustness to lower-SSI inputs in this segmentation-free OCT-based VF framework.

## Introduction

Estimating functional visual field (VF) sensitivity from structural optical coherence tomography (OCT) data has become an active area of glaucoma research. Various analytical approaches, including machine learning and deep learning, have been used to estimate either global indices or pointwise VF measurements from OCT-derived inputs. These studies have used a variety of OCT-derived inputs, including segmentation-derived retinal nerve fiber layer or ganglion cell thickness measurements/maps, image-based OCT representations, and, more recently, unsegmented three-dimensional OCT volumes.^1^^–^^16^

A common methodological feature across this literature is the exclusion of scans below a device-specific image-quality threshold before model development or evaluation. Studies based on the CIRRUS platform (Carl Zeiss Meditec, Jena, Germany) have commonly used signal strength thresholds around 6,^1^^–^^7^^,^^9^ Topcon (Tokyo, Japan)-based studies have used image-quality thresholds around 40,^7^^,^^8^ studies based on SPECTRALIS imaging (Heidelberg Engineering, Heidelberg, Germany) have used quality-score thresholds around 15,^9^^–^^12^ and NIDEK (Aichi, Japan)-based studies have commonly used signal strength index (SSI) thresholds around 7.^13^^–^^16^ Although the specific metric and cutoff vary by manufacturer, the underlying assumption has generally been the same: Scans of lower image quality should be excluded before model development or evaluation.

This assumption is well grounded for conventional structure–function analysis. When quantitative parameters depend on automated segmentation of retinal layers, reduced signal strength increases the likelihood of segmentation errors and can destabilize thickness measurements.^17^^,^^18^ Signal strength can also affect measured retinal thickness even when segmentation is successful, thereby introducing additional variability into structure–function analyses.^18^^–^^20^ Excluding low-quality scans has therefore been a reasonable strategy when analyses rely on segmentation-derived features.

Whether the same logic should be applied to deep learning–based VF estimation, however, remains uncertain. In routine clinical practice, OCT image quality varies substantially across patients and visits. Restricting model development to high-quality scans may improve internal consistency, but it may also reduce exposure to the range of inputs encountered in real-world care. This question may be particularly relevant for segmentation-free models that operate directly on raw OCT volumes rather than on segmentation-derived measurements.

In our previous work, training on a more inclusive dataset yielded better VF estimation performance than training restricted to eyes with glaucoma.^15^ Extending that concept to OCT image quality, it remains unclear whether excluding low-SSI scans during training is advantageous for OCT-based VF estimation. For NIDEK systems in particular, SSI ≥ 7 is often used as a practical quality threshold,^13^^–^^16^ but it is unknown whether restricting training to scans meeting this criterion improves performance or whether including lower-SSI scans instead improves robustness under suboptimal imaging conditions.

Accordingly, in this multicenter retrospective study, we directly compared two training strategies for a three-dimensional convolutional neural network (3DCNN) that estimates VF from macular OCT volumes acquired with NIDEK devices: a pragmatic all-available-data strategy that used scans across the full SSI range and a conventional SSI-filtered strategy that used only scans with SSI ≥ 7. Both models were evaluated on identical validation and test datasets spanning the full SSI range. Because the primary training datasets differed in both sample size and SSI distribution, this comparison was designed to evaluate the practical consequence of applying an SSI-based training exclusion rule, rather than to determine the specific contribution of low-SSI exposure independent of training-set size and overall training-set composition. We hypothesized that the all-SSI training strategy would be associated with improved performance in low-SSI test samples without materially degrading performance in higher-SSI samples.

## Methods

### Study Design and Participants

This retrospective multicenter study was approved by the Institutional Review Board of Shimane University Hospital (IRB No. KS20230719-3), the Institutional Review Board of Jichi Medical University (IRB No. Rindai24-130), and the corresponding ethics committees at the participating institutions. The study adhered to the tenets of the Declaration of Helsinki. Participating institutions were Shimane University Hospital (Shimane, Japan), Jichi Medical University (Tochigi, Japan), Teikyo University Hospital (Tokyo, Japan), JCHO Tokyo Shinjuku Medical Center (Tokyo, Japan), and Omiya Hamada Eye Clinic West Entrance Branch (Saitama, Japan).

Given the retrospective design, the requirement for written informed consent was waived at each site. Instead, an opt-out approach was used, and study information was publicly disclosed at each institution to allow patients to decline participation. Clinical data were retrospectively collected from patients who underwent macular OCT imaging and/or Humphrey Field Analyzer (HFA; Carl Zeiss Meditec) testing during routine care between March 15, 2010, and July 9, 2025. To reflect real-world clinical practice, no exclusions were made on the basis of ocular diagnosis. This approach was also informed by our previous work, in which training on a more inclusive dataset yielded better VF estimation performance than training restricted to eyes with glaucoma.^15^ Diagnosis labels were not uniformly available across institutions; therefore, diagnosis-based stratification and diagnosis-restricted sensitivity analyses were not performed.

### Inclusion Criteria

For initial data extraction, eyes were eligible if they had at least one macular OCT scan or at least one HFA examination performed with the 30-2, 24-2, or 10-2 pattern using the Swedish Interactive Threshold Algorithm (SITA) Standard strategy.

### Data Acquisition and Pairing

Macular OCT images were acquired with NIDEK devices (RS-3000 series and Mirante) using a 9 × 9-mm volumetric scan centered on the macula. All scans were retained irrespective of pupil dilation status. For each OCT scan, the temporally closest HFA examination within ±90 days was selected. OCT scans without an HFA examination within this window were excluded. When multiple eligible examinations were available for the same eye, all qualifying pairs of OCT and HFA examinations were retained. For 30-2 HFA examinations, peripheral test locations not shared with the 24-2 pattern were removed to align the spatial configuration with the 24-2 grid. No SSI-based exclusion was applied during dataset construction. Visual field reliability filtering was limited to false-positive responses; tests with a false-positive rate of 15% or greater were excluded. False-negative responses and fixation losses were not used as exclusion criteria. All remaining data were retained to preserve real-world variability.

### Model Development and Training Strategy

**Figure 1.**
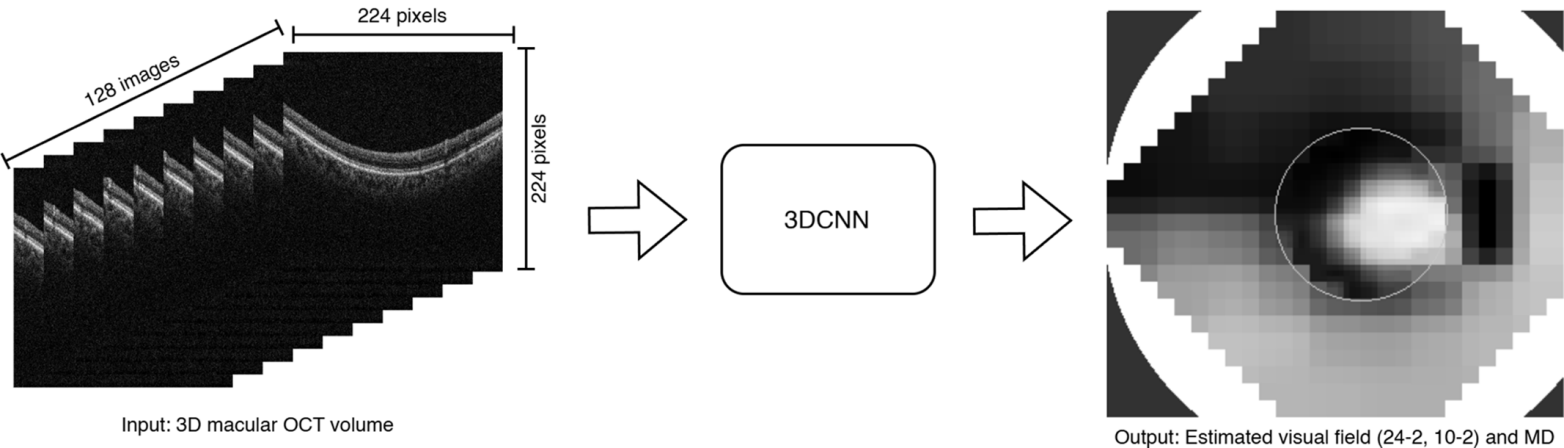
Overview of the three-dimensional OCT-based visual field estimation model. A three-dimensional macular OCT volume (224 × 224 × 128) was used as input to a 3DCNN. The model directly estimated pointwise visual field sensitivities for the 24-2 and 10-2 test patterns, along with the corresponding MD values, without segmentation. The *right panel* shows an example of the estimated visual field.

A 3DCNN was trained to estimate VF measurements from OCT volumes. The architecture was based on EfficientNet 3D-b0^21^^–^^23^ and incorporated an additional dropout layer (dropout rate, 30%) before the final fully connected layer as a standard regularization procedure. The network was trained from scratch without fine-tuning. Input OCT volumes were resampled to 224 × 224 × 128 voxels and normalized using *z*-score normalization. To standardize laterality, all left-eye OCT volumes were horizontally flipped to match right-eye orientation before model input. The corresponding 24-2 and 10-2 pointwise VF labels were remapped by horizontal reflection of the test-location grid, whereas mean deviation (MD) values were left unchanged. The network directly output pointwise sensitivities for the 24-2 pattern (52 locations), 24-2 MD, pointwise sensitivities for the 10-2 pattern (68 locations), and 10-2 MD through the final fully connected layer (122 outputs in total) (Fig. 1).

Vertical flipping was used for data augmentation, as described in previous studies.^14^^–^^16^ During training, vertically flipped copies of the training samples were added to the training set, such that the augmented training set contained both the original and vertically flipped orientations. For vertically flipped OCT volumes, the corresponding 24-2 and 10-2 pointwise VF labels were remapped by superior–inferior reflection of the test-location grid, whereas MD values were left unchanged. At test time, estimates were obtained from both the original OCT volume and its vertically flipped version. Estimates from the vertically flipped input were remapped back to the original orientation by applying the inverse superior–inferior reflection to the pointwise VF outputs, whereas MD outputs were left unchanged. The final test-time output was calculated by averaging the original and remapped vertically flipped estimates.

Because not all OCT scans were paired with both 24-2 and 10-2 VF measurements, target masking was used to handle missing output labels. For each training sample, observed targets were assigned a mask value of 0, whereas missing targets were assigned a mask value of 1. If a 24-2 VF measurement was absent, all 53 corresponding 24-2 outputs, consisting of 52 pointwise sensitivities and 24-2 MD, were masked. Similarly, if a 10-2 VF measurement was absent, all 69 corresponding 10-2 outputs, consisting of 68 pointwise sensitivities and 10-2 MD, were masked. During training, mean squared error was calculated only over observed targets by applying the target mask during loss calculation, equivalently multiplying the squared error by 1 − mask and averaging over unmasked elements. No label imputation was performed.

Model training used the Adam optimizer with a mini-batch size of 4. Mean squared error served as the loss function. Training was run for a fixed seven epochs rather than being terminated by early stopping. The learning rate was initialized at 6.0 × 10^−4^, increased to 1.0 × 10^−3^ over the first two epochs, and then decreased to 6.0 × 10^−4^ over the subsequent five epochs. After completion of the seven epochs, the checkpoint with the lowest validation loss was selected as the best epoch and used for inference on the held-out test set. Target values were used as recorded without clipping or exclusion. Validation and test metrics were also computed only for observed ground-truth labels with mask value 0; missing labels were excluded from metric calculation and were not imputed. The network generated raw estimates for both pointwise sensitivity and MD; only pointwise sensitivity estimates below 0 dB were set to 0 dB, whereas MD estimates were left unchanged.

Two training strategies were compared. The all-SSI-trained model used all available training OCT scans irrespective of SSI. The SSI ≥ 7-trained model used only training scans with SSI ≥ 7, consistent with conventional practice.^13^^–^^16^ Validation and test sets were identical between strategies and included all eligible samples irrespective of SSI. The primary comparison therefore evaluated the practical consequence of applying an SSI-based training exclusion rule, comparing a pragmatic all-available training-data strategy with conventional SSI-based filtering. Because the all-SSI-trained model retained SSI < 7 training scans but the SSI ≥ 7-trained model excluded them, the two primary training sets differed in both sample size and SSI distribution; therefore, this comparison was not intended to determine the specific contribution of low-SSI exposure independent of training-set size and overall training-set composition.

To further examine the roles of low-SSI exposure and training-set size, we performed two complementary analyses. First, in an additive low-SSI exposure analysis, all SSI ≥ 7 training scans were retained, and 25% or 50% of the available SSI < 7 training scans were added to the training set. Low-SSI scans were subsampled separately within the 24-2-paired and 10-2-paired training data while preserving the SSI distribution as closely as possible. The 0% and 100% low-SSI exposure conditions corresponded to the SSI ≥ 7-trained model and the all-SSI-trained model, respectively. Validation and test sets were kept identical across all additive exposure conditions. This analysis was descriptive.

Second, as a size-matched sensitivity analysis, we additionally trained a size-matched all-SSI-trained model. For this analysis, the all-SSI training set was downsampled separately for the 24-2-paired and 10-2-paired training data to exactly match the number of training samples in the SSI ≥ 7-trained model while preserving the original MD distribution as closely as possible by systematic subsampling after sorting by MD. Validation and test sets were identical to those used in the primary analysis.

### Cross-Validation Procedure

Model development and evaluation used patient-wise 10-fold cross-validation to prevent data leakage. In each iteration, eight folds were used for training, one for validation, and one for testing, and the process was repeated until every fold had served as the test set. For each iteration, the model from the epoch with the best validation performance was selected and evaluated on the held-out test set. To balance case mix across folds, patients were stratified by institution, test pattern, disease severity (defined by the median MD), and the number of HFA examinations per patient (dichotomized at the median).

### Outcome Measures

Performance was assessed using four prespecified endpoints: pointwise mean absolute error (MAE) for the 24-2 and 10-2 patterns and absolute error of MD for the two patterns. These endpoints were chosen to capture both local and global accuracy. For validation and test evaluation, each endpoint was calculated only for observed ground-truth labels with mask value 0; missing labels with mask value 1 were excluded from metric calculation, and no label imputation was performed.

### Model Comparison and Statistical Analysis

Model performance was compared within two SSI-defined subgroups: SSI < 7 and SSI ≥ 7. The primary analysis used patient-level cluster bootstrap resampling with 10,000 iterations, with patient ID as the resampling unit to account for within-patient correlation. For each endpoint, we estimated the mean between-model difference in error and its 95% confidence interval (CI). Differences were defined as the error of the all-SSI-trained model minus that of the SSI ≥ 7-trained model; negative values therefore indicate better performance of the all-SSI-trained model. Bonferroni correction was applied across the eight prespecified comparisons.

As a sensitivity analysis addressing OCT-HFA temporal mismatch, we repeated the primary model comparison after restricting the held-out test samples to OCT-HFA pairs acquired within ±30 days. The same trained models, SSI subgroups, endpoints, and patient-level cluster bootstrap framework were used.

For an additional sensitivity analysis addressing VF reference-standard reliability, we repeated the primary model comparison after restricting the held-out test samples to VFs with false-positive responses < 15%, false-negative responses < 15%, and fixation losses < 15%. The same trained models, SSI subgroups, four prespecified endpoints, and patient-level cluster bootstrap framework were used. Bonferroni correction was applied across the eight comparisons.

An additional sensitivity analysis addressed heterogeneity in the source visual-field test pattern. We repeated the 24-2 model comparison after restricting the held-out test samples to native 24-2 visual fields and excluding 30-2 examinations that had been truncated to the 24-2 grid. The same trained models, SSI subgroups, 24-2 endpoints, and patient-level cluster bootstrap framework were used. Bonferroni correction was applied across the four comparisons.

As an additional device-specific sensitivity analysis, NIDEK devices were grouped into RS-3000 series devices (RS-3000, RS-3000 Advance 2, and RS-330) and Mirante. Within the SSI < 7 test subgroup, model performance was summarized separately for each device family using the same four endpoints and patient-level cluster bootstrap framework as in the primary analysis. Holm correction was applied across the eight device-family-specific comparisons.

To formally assess whether the SSI-dependent between-model difference varied by device family, we performed a device-by-SSI interaction analysis. The paired error difference was defined as the error of the all-SSI-trained model minus that of the SSI ≥ 7-trained model. Linear models were fitted with the paired error difference as the dependent variable and SSI subgroup, device family, and their interaction as explanatory variables. Cluster-robust standard errors were computed with patient ID as the clustering variable. Holm correction was applied across the four endpoint-specific interaction tests. As a supplementary analysis, mean performance was calculated for each of the 10 matched test folds, and paired comparisons between models were performed with the Wilcoxon signed-rank test.

To evaluate whether the between-model difference depended on SSI subgroup, we performed an interaction analysis. For each test sample, the paired error difference between models was calculated, and a linear model was fitted with this difference as the dependent variable and SSI subgroup as the explanatory variable. Cluster-robust standard errors were computed with patient ID as the clustering variable. Bonferroni correction was applied across the 12 reported terms and contrasts.

In a supplementary sensitivity analysis, the size-matched all-SSI-trained model was compared with the SSI ≥ 7-trained model using the same patient-level cluster bootstrap framework. To maintain direct correspondence with the primary analysis, performance was evaluated within the same two SSI-defined subgroups and across the same four prespecified endpoints; Bonferroni correction was applied across the eight corresponding comparisons.

An additional sensitivity analysis addressed potential confounding by disease severity within the low-SSI subgroup. We performed an MD-stratified model comparison in the SSI < 7 test subgroup. HFA MD was categorized into three severity strata: mild (MD > −6 dB), moderate (−12 dB < MD ≤ −6 dB), and severe (MD ≤ −12 dB). Within each severity stratum, the all-SSI-trained model was compared with the SSI ≥ 7-trained model. Patient-level cluster bootstrap resampling with 10,000 iterations was used, and Bonferroni correction was applied across the 12 comparisons, comprised of four endpoints across three severity strata.

### Assessment of SSI-Dependent Systematic Bias

To assess whether model errors showed systematic SSI-dependent bias, we examined the association between SSI and signed error, defined as the OCT-based estimate minus the measured HFA value. For each endpoint, a linear regression model was fitted with signed error as the dependent variable and SSI as the explanatory variable.

To account for repeated measurements within patients, patient-level cluster bootstrap resampling with 10,000 iterations was used. In each bootstrap sample, patients were resampled with replacement, and all corresponding observations were retained. The regression slope was estimated for each bootstrap sample, and 95% CIs were derived from the bootstrap distribution. A slope was considered statistically significant if the CI excluded zero.

In additional severity-stratified analyses, the association between SSI and signed error was evaluated within mild, moderate, and severe strata. Severity was first defined by HFA MD as mild (MD > −6 dB), moderate (−12 dB < MD ≤ −6 dB), and severe (MD ≤ −12 dB). As an exploratory sensitivity analysis, the same analysis was repeated using OCT-based estimated visual field (OCT-VF) MD rather than HFA MD to define the severity strata. Bonferroni correction was applied across the 12 severity-stratified comparisons for each stratification approach.

### Assessment of the Relationship Between SSI and Visual Field Status

To characterize the relationship between OCT image quality and VF status, HFA measurements were stratified by SSI. Distributions of pointwise sensitivity and MD were summarized with boxplots at each SSI level, and linear regression was used to quantify the association between SSI and HFA measurements.

### Assessment of the Relationship Between HFA Measurements and OCT-Based VF Estimates

To evaluate the relationship between measured HFA values and OCT-VF, analyses were performed using the all-SSI-trained model. Agreement was first summarized using MAE and mean error (ME) for 24-2 and 10-2 pointwise sensitivities and MD. In addition, for both test patterns and for both pointwise sensitivity and MD, OCT-VF estimates were stratified according to the corresponding HFA values in 1-dB bins and summarized using boxplots. For pointwise sensitivity, these analyses were performed both after pooling all test locations within each pattern and separately for each individual test location in the 24-2 (52 locations) and 10-2 (68 locations) test patterns.

### Implementation Details

All statistical tests were two sided. Analyses were performed in Python 3.12.9 using statsmodels 0.14.5, SciPy 1.16.1, and PyTorch 2.7.0. Model training and inference were performed on a GeForce RTX 5090 GPU (NVIDIA, Santa Clara, CA). For the additive low-SSI exposure analysis, the 25% and 50% low-SSI subsamples were generated once for each cross-validation fold and were not repeated across multiple random subsamples. The same patient-level fold assignments were used for all training strategies to ensure identical validation and held-out test sets across models. Patient assignment to folds used random seed 42; no additional fixed random seed was imposed for low-SSI subsampling or other stochastic model-training processes. The EfficientNet 3D implementation was based on the publicly available EfficientNet-PyTorch-3D repository.^23^ The overall workflow for dataset construction, HFA reliability filtering, and pairing of OCT and HFA examinations is shown in Supplementary Figure S1.

## Results

### Dataset Characteristics

The dataset was comprised of 79,803 paired OCT and HFA examinations from 8511 patients (16,389 eyes), including 57,520 24-2 pairs and 22,283 10-2 pairs. The mean number of OCT examinations per eye was 4.87 ± 6.63. Mean MD was −8.8 ± 8.8 dB for 24-2 and −11.6 ± 10.0 dB for 10-2, indicating a wide range of functional loss. Mean age was 66.2 ± 15.6 years. Mean SSI was 8.03 ± 1.65, and 85.5% of scans had SSI ≥ 7 (Table).

**Table.** Dataset Characteristics

Characteristic	Value
Total patients, *n*	8,511
Patients in 24-2, *n*	8,244
Patients in 10-2, *n*	2,509
Total eyes, *n*	16,389
Eyes in 24-2, *n*	15,839
Eyes in 10-2, *n*	4,564
Total paired examinations, *n*	79,803
Paired examinations in 24-2, *n*	57,520
Paired examinations in 10-2, *n*	22,283
Paired data per eye, mean ± SD	4.87 ± 6.63
MD of the HFA 24-2 (dB), mean ± SD	−8.8 ± 8.8
MD of the HFA 10-2 (dB), mean ± SD	−11.6 ± 10.0
Age (y), mean ± SD	66.2 ± 15.6
SSI, mean ± SD	8.03 ± 1.65
Percentage of scans with SSI ≥ 7	85.5

The 24-2 and 10-2 counts are not mutually exclusive.

### SSI Distribution

**Figure 2.**
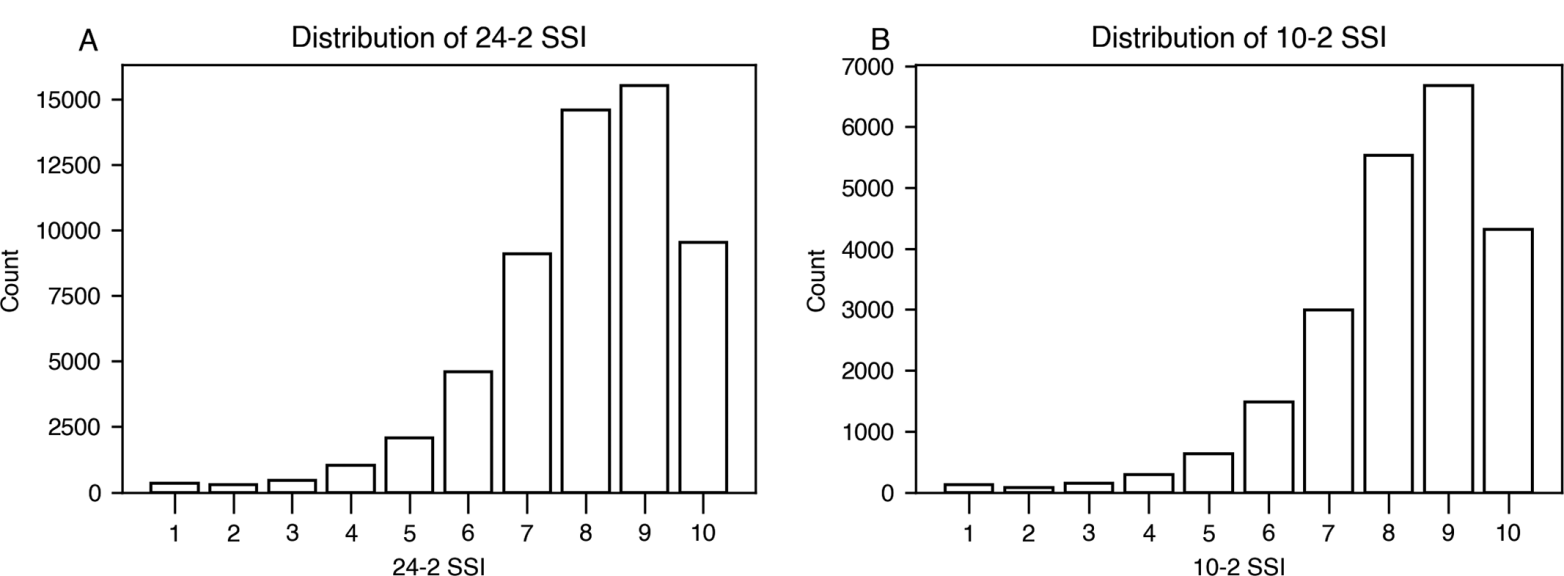
Distribution of OCT SSIs in the study dataset. (**A**, **B**) Histograms show SSI distributions for datasets paired with HFA 24-2 (**A**) and HFA 10-2 (**B**). SSI values were broadly distributed, with most scans clustering around 8 to 9. Lower-SSI scans (SSI < 7) were also well represented, whereas very low SSI values (≤ 3) were uncommon. The absence of a distinct separation at SSI = 7 indicates that image quality was distributed continuously rather than dichotomously.

SSI values were broadly distributed in both the 24-2-paired and 10-2-paired datasets, with a peak around 8 to 9 (Fig. 2). Although most scans had relatively high SSI values, lower-SSI scans, including those with SSI < 7, were well represented, whereas very low SSI values (≤3) were uncommon. The distribution showed no clear separation around SSI = 7, supporting the view that image quality in this dataset was continuous rather than dichotomous.

### Performance Comparison Between Training Strategies

**Figure 3.**
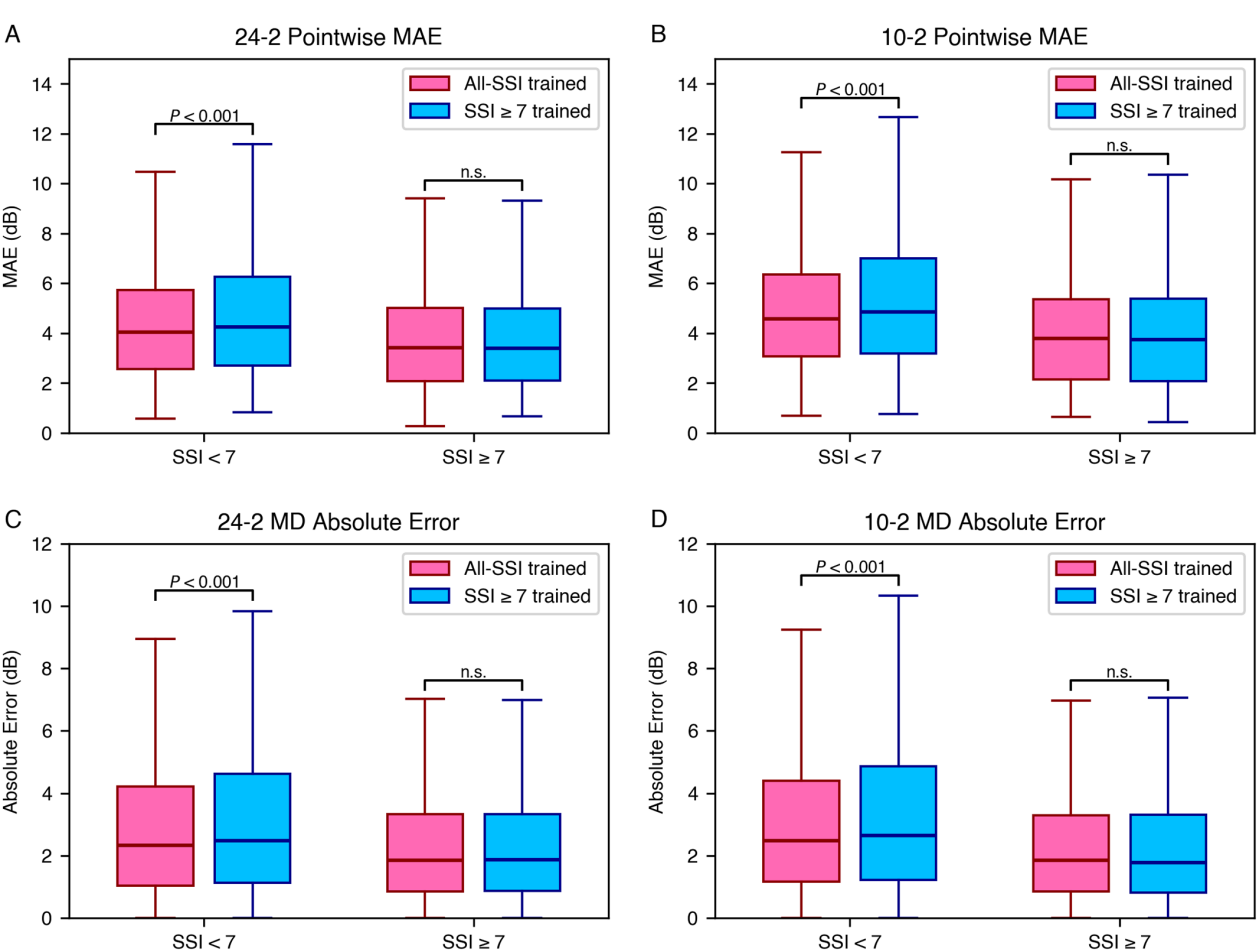
Comparison of model performance between training strategies across SSI subgroups. Boxplots show errors for the all-SSI-trained model and the SSI ≥ 7-trained model within the SSI < 7 and SSI ≥ 7 subgroups. (**A–D**) Panels show 24-2 pointwise MAE (**A**), 10-2 pointwise MAE (**B**), 24-2 MD absolute error (**C**), and 10-2 MD absolute error (**D**). The held-out test subgroup sizes were as follows: for 24-2, 48,732 samples from 7771 patients in the SSI ≥ 7 subgroup and 8788 samples from 3506 patients in the SSI < 7 subgroup; for 10-2, 19,523 samples from 2390 patients in the SSI ≥ 7 subgroup and 2760 samples from 1037 patients in the SSI < 7 subgroup. In the SSI < 7 subgroup, the all-SSI-trained model had significantly lower errors for all endpoints (all adjusted *P* < 0.001, patient-level cluster bootstrap) (Supplementary Table S1). No significant between-model differences were observed in the SSI ≥ 7 subgroup. n.s., not significant.

In the primary patient-level cluster bootstrap analysis, errors within the SSI ≥ 7 subgroup were similar between the two models for all four endpoints, with no statistically significant differences after Bonferroni correction (Fig. 3, Supplementary Table S1). By contrast, within the SSI < 7 subgroup, the all-SSI-trained model yielded significantly lower errors for all endpoints. Relative to the SSI ≥ 7-trained model, the all-SSI-trained model reduced error by 0.439 dB for 24-2 pointwise MAE, 0.417 dB for 24-2 MD absolute error, 0.473 dB for 10-2 pointwise MAE, and 0.453 dB for 10-2 MD absolute error (all adjusted *P* < 0.001) (Supplementary Table S1).

Restricting the evaluation set to OCT-HFA pairs acquired within ±30 days did not materially change the primary findings: The all-SSI-trained model remained more accurate in the SSI < 7 subgroup, whereas no significant difference was observed in the SSI ≥ 7 subgroup (Supplementary Table S2). Restricting the evaluation set to VFs with false-positive responses < 15%, false-negative responses < 15%, and fixation losses < 15% did not materially change the primary findings. The all-SSI-trained model remained more accurate in the SSI < 7 subgroup across all four endpoints, whereas no significant difference was observed in the SSI ≥ 7 subgroup (Supplementary Table S3).

Excluding 30-2 examinations that had been truncated to the 24-2 grid did not materially change the 24-2 findings. In the native 24-2-only sensitivity analysis, the all-SSI-trained model remained more accurate in the SSI < 7 subgroup for both 24-2 pointwise MAE and 24-2 MD absolute error, whereas no significant difference was observed in the SSI ≥ 7 subgroup (Supplementary Table S4).

Device-family-specific analyses showed that the direction of the between-model difference in the SSI < 7 subgroup was consistent across RS-3000 series and Mirante devices, with lower errors for the all-SSI-trained model in both device families (Supplementary Table S5). However, the Mirante low-SSI subgroup was small, particularly for 10-2 outcomes. Formal device-by-SSI interaction analyses did not show statistically significant interaction effects after Holm correction across the four endpoints (Supplementary Table S6).

A supplementary fold-wise analysis using the Wilcoxon signed-rank test showed the same pattern: no significant differences within the SSI ≥ 7 subgroup and significantly lower errors for the all-SSI-trained model within the SSI < 7 subgroup across all four endpoints (Supplementary Table S7). To further describe performance across progressively increasing low-SSI exposure, we performed an additive low-SSI exposure analysis in which all SSI ≥ 7 training scans were retained and 25%, 50%, or 100% of SSI < 7 training scans were added. In this descriptive analysis, errors generally decreased as the proportion of added low-SSI scans increased, and the all-SSI-trained model showed the lowest errors across all four endpoints. Compared with SSI ≥ 7-only training, adding 25%, 50%, and 100% of low-SSI scans reduced 24-2 pointwise MAE by 0.349, 0.411, and 0.440 dB, respectively, and reduced 10-2 pointwise MAE by 0.415, 0.437, and 0.473 dB, respectively. Similar overall reductions were observed for 24-2 and 10-2 MD absolute errors, although the 10-2 MD error showed a small nonmonotonic fluctuation between the 25% and 50% low-SSI exposure conditions (Supplementary Table S8).

In an additional size-matched sensitivity analysis, the size-matched all-SSI-trained model remained more accurate than the SSI ≥ 7-trained model in the SSI < 7 subgroup across all four endpoints, with reductions of 0.390 dB for 24-2 pointwise MAE, 0.372 dB for 24-2 MD absolute error, 0.482 dB for 10-2 pointwise MAE, and 0.454 dB for 10-2 MD absolute error (all adjusted *P* < 0.001) (Supplementary Table S9). By contrast, in the SSI ≥ 7 subgroup, errors were modestly but significantly higher across all four endpoints in the size-matched all-SSI-trained model (difference range, 0.054–0.092 dB; all adjusted *P* ≤ 0.002).

### MD-Stratified Model Comparison

To address potential confounding by disease severity within the low-SSI subgroup, we performed an additional MD-stratified analysis in the SSI < 7 test subgroup. HFA MD was categorized as mild (MD > −6 dB), moderate (−12 dB < MD ≤ −6 dB), or severe (MD ≤ −12 dB). Across all severity strata and endpoints, the all-SSI-trained model showed lower point estimates of error than the SSI ≥ 7-trained model. The differences were most consistent in the moderate and severe strata, whereas estimates in the mild stratum were smaller and less stable, particularly for 10-2 outcomes. These findings suggest that the advantage of all-SSI training in low-SSI test data was not solely attributable to the overrepresentation of advanced VF loss in the low-SSI subgroup (Supplementary Table S10).

### Interaction Analysis

Interaction analysis confirmed that the between-model difference depended on SSI subgroup. For all endpoints, the additional change associated with SSI < 7 was significantly negative (Supplementary Table S11), indicating that the advantage of the all-SSI-trained model was substantially greater in low-SSI than in higher-SSI data.

### Association Between SSI and Error

**Figure 4.**
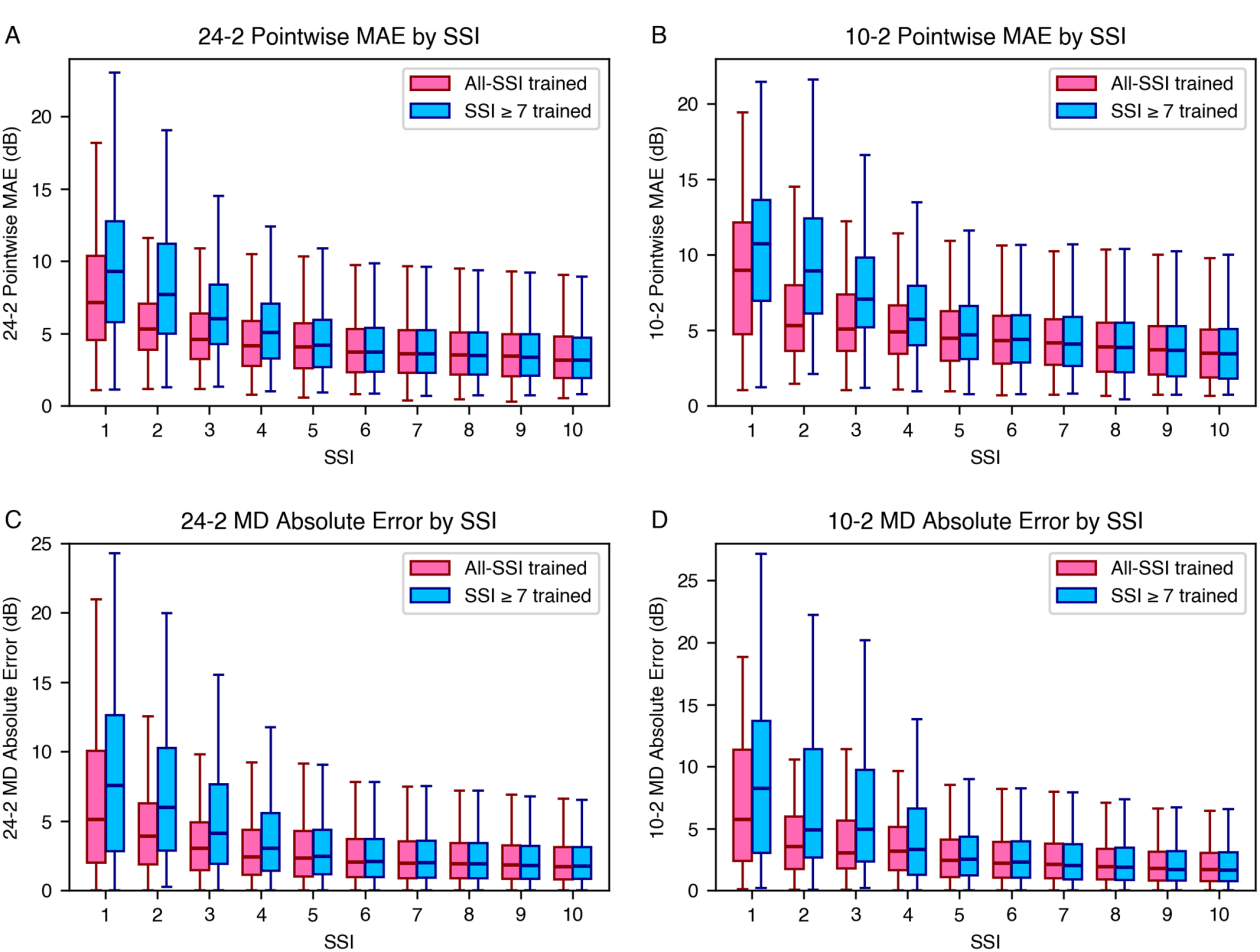
Relationship between SSI and estimation error. Boxplots show errors across SSI levels for the all-SSI-trained model and the SSI ≥ 7-trained model. (**A–D**) Panels show 24-2 pointwise MAE (**A**), 10-2 pointwise MAE (**B**), 24-2 MD absolute error (**C**), and 10-2 MD absolute error (**D**). For both models, error increased as SSI decreased, without an evident threshold effect at SSI = 7. The all-SSI-trained model generally showed lower errors than the SSI ≥ 7-trained model, particularly at lower SSI values.

When SSI was treated as a continuous variable, error increased progressively as SSI decreased for all endpoints (Fig. 4). This deterioration was gradual and did not show a threshold-like change at SSI = 7. Across the SSI spectrum, the all-SSI-trained model generally had lower errors than the SSI ≥ 7-trained model, with the gap widening as SSI decreased. In the SSI ≥ 7-trained model, errors were relatively stable at moderate SSI values but increased at very low SSI values (approximately SSI ≤ 3).

### SSI-Dependent Systematic Bias

**Figure 5.**
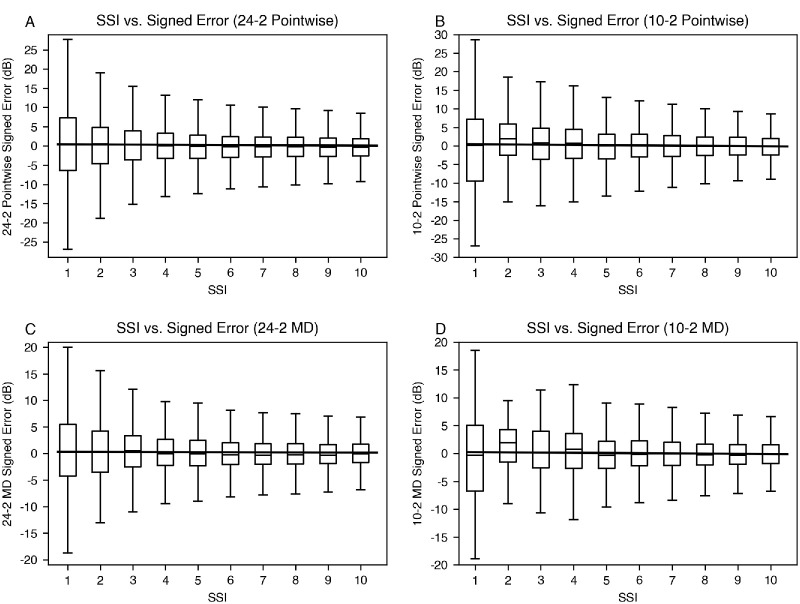
Relationship between SSI and signed error. (**A–D**) Boxplots show signed errors (OCT-based estimates minus HFA measurements) across SSI levels for 24-2 pointwise sensitivity (**A**), 10-2 pointwise sensitivity (**B**), 24-2 MD (**C**), and 10-2 MD (**D**). Positive values indicate overestimation by the model, and negative values indicate underestimation. Across endpoints, median signed error remained close to zero throughout the SSI range. *Solid lines* indicate linear regression fits. Bootstrap-based 95% CIs for all regression slopes included zero (Supplementary Table S12).

Across all endpoints, median signed error remained close to zero across the full SSI range (Fig. 5), with no obvious overall directional shift toward overestimation or underestimation at lower SSI values. Consistent with this visual impression, unstratified regression slopes relating SSI to signed error were small, and all 95% CIs included zero (Supplementary Table S12). Thus, in the overall unstratified analysis, there was no statistically significant monotonic association between SSI and signed error.

Severity-stratified analyses based on HFA MD showed that the association between SSI and signed error varied across the VF damage range (Supplementary Table S13). Mild eyes showed positive slopes, indicating more negative signed errors at lower SSI values, whereas severe eyes tended to show negative slopes. As an exploratory sensitivity analysis, we repeated the severity-stratified signed-error analysis using OCT-VF MD, rather than HFA MD, to define mild, moderate, and severe strata (Supplementary Table S14). In this analysis, slopes were generally small, and none remained statistically significant after Bonferroni correction.

### Association Between SSI and Visual Field Measurements

Lower SSI values were associated with worse VF measurements (Supplementary Fig. S2). Both pointwise sensitivity and MD increased with higher SSI in the 24-2 and 10-2 datasets. Linear regression showed positive associations ranging from 0.72 to 1.04 dB per unit increase in SSI.

### Model Performance

Performance of the all-SSI-trained model on the full dataset is summarized in Supplementary Table S15. Mean absolute error was 3.99 dB for 24-2 pointwise sensitivity, 4.22 dB for 10-2 pointwise sensitivity, 2.62 dB for 24-2 MD, and 2.59 dB for 10-2 MD.

**Figure 6.**
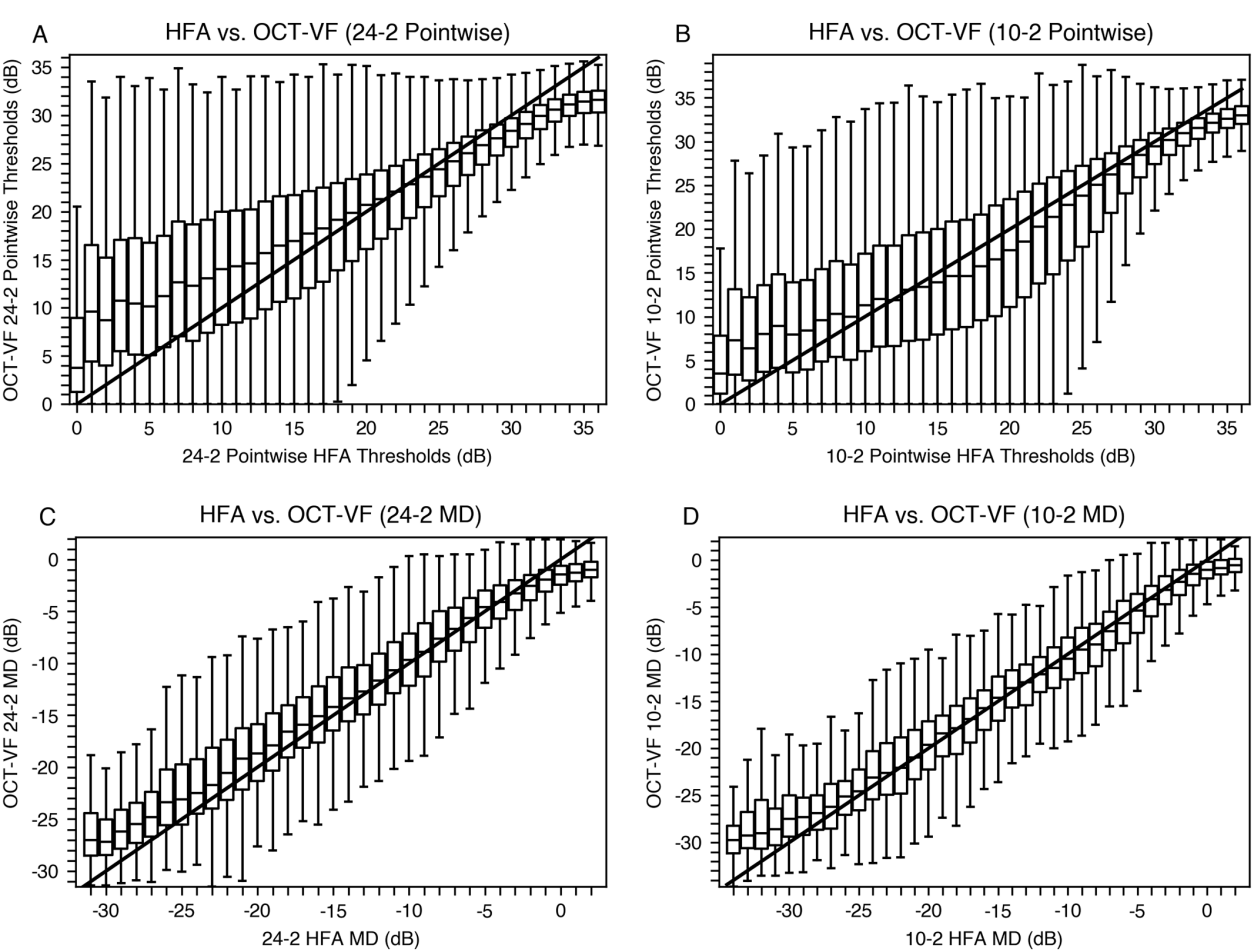
Relationship between HFA measurements and OCT-VF. Boxplots show the distribution of OCT-VF values for each corresponding HFA value for 24-2 pointwise sensitivity (**A**), 10-2 pointwise sensitivity (**B**), 24-2 MD (**C**), and 10-2 MD (**D**). The diagonal line represents the line of identity (y = x). Estimates were generated by the all-SSI-trained model and evaluated on the full dataset. Across endpoints, OCT-VF closely tracked HFA measurements over a broad dynamic range, with greater variability at lower sensitivity values.

The relationship between OCT-VF estimates and corresponding HFA measurements is shown in Figure 6 and Supplementary Figures S3 and S4. Across endpoints, OCT-VF estimates closely tracked HFA measurements over a broad dynamic range, although variability increased at lower sensitivity levels. Pointwise analyses showed a similar pattern in the 24-2 and 10-2 grids, with greater dispersion in advanced VF loss. A spatial pattern was also apparent: Deviations from HFA tended to be smaller at test locations farther from the optic disc (nasal VF locations), whereas locations closer to or beyond the optic disc showed larger deviations.

## Discussion

In this multicenter study, we examined a methodological assumption that has been widely embedded in prior OCT-based VF estimation studies: that lower-quality OCT scans should be excluded before model development. The principal finding was that, compared with SSI-based filtering, training on all available scans yielded better performance in low-SSI test data without materially affecting performance in scans with SSI ≥ 7. Because approximately 15% of examinations in our dataset had SSI < 7, this issue is relevant to routine clinical practice rather than to a rare edge case. In this setting, the key question is not whether a scan satisfies a predefined quality cutoff, but whether its inclusion helps the model learn structure–function relationships that remain stable across the range of data encountered in real-world care. Viewed in this way, routine exclusion of low-SSI scans did not improve performance in this segmentation-free 3D OCT-VF framework and may reduce robustness to lower-quality scans within this dataset.

This interpretation differs in emphasis from conventional OCT quality filtering. Most prior OCT-VF studies have been developed using quality-filtered datasets with device-specific thresholds applied during dataset construction.^1^^–^^16^ For NIDEK systems, SSI around 7 has commonly been used as a practical quality criterion.^13^^–^^16^ However, such thresholds were established primarily in the context of measurement reliability and segmentation-derived structural analysis, where reduced signal strength can increase segmentation errors and alter thickness measurements even when segmentation is nominally successful.^17^^–^^20^ Our findings do not dispute the value of such filtering for segmentation-dependent analyses; rather, they suggest that the same logic may not directly apply to segmentation-free 3D models that learn from raw OCT volumes. More broadly, the results highlight a general issue in ophthalmic artificial intelligence: Highly curated datasets may improve internal consistency, but they can also narrow the training distribution and reduce robustness when models encounter the variability of routine clinical data.

Several aspects of our results support this conclusion. First, SSI behaved as a continuous modifier of estimation difficulty rather than a binary indicator of scan usability. In our dataset, SSI values were continuously distributed and showed no natural separation around 7. Consistently, estimation error worsened gradually as SSI decreased, rather than showing a threshold-like change at SSI = 7. This graded pattern argues against using SSI = 7 as an absolute eligibility criterion for model development in this setting.

Second, differences between the two training strategies were concentrated in lower-SSI data and became larger as SSI decreased. The SSI ≥ 7-trained model performed similarly to the all-SSI-trained model at moderate and high SSI levels, but its errors increased more sharply at lower SSI values, whereas the all-SSI-trained model showed a less pronounced increase in error as SSI decreased. Although the absolute error reductions in the SSI < 7 subgroup were modest—approximately 0.4 to 0.5 dB relative to a full-dataset pointwise MAE of approximately 4 dB—they were consistent across endpoints and sensitivity analyses and were observed in the subgroup likely to be most susceptible to image-quality degradation and HFA reference-standard variability. One plausible explanation is that exposure to a wider spectrum of scan qualities enabled the network to learn features that remained informative despite reduced signal quality. This interpretation was supported by the supplementary size-matched analysis, in which the low-SSI advantage persisted after matching training-set size. However, the same analysis showed a small but statistically significant worsening in SSI ≥ 7 data, consistent with the reduced number of higher-SSI training samples. Thus, the findings support a contribution of training-set composition to low-SSI robustness while indicating that high-SSI training exposure should not be reduced without cost. For routine-care deployment, the primary all-available-data comparison may be more clinically relevant because including low-SSI scans does not require discarding high-SSI scans.

Third, in the overall unstratified analysis, the improved performance of the all-SSI-trained model in low-SSI data was not accompanied by a statistically significant monotonic association between SSI and signed error. Signed errors remained centered near zero across the SSI range, and no clear overall directional shift toward overestimation or underestimation was observed as SSI decreased. This finding differs from prior reports showing that reduced OCT signal quality can systematically influence segmentation-derived structural measurements.^18^^–^^20^ One possible explanation is that the present framework operated directly on raw OCT volumes rather than on segmentation-derived thickness maps, which are particularly vulnerable to signal-related artifacts. In addition, the large multicenter dataset may have helped the network distinguish acquisition-related attenuation and noise from disease-related structural change.

However, severity-stratified analyses provided a more nuanced picture. When severity was defined by HFA MD, small SSI-related signed-error slopes were observed, with positive slopes in mild eyes and negative slopes in severe eyes. In contrast, when severity strata were defined by OCT-VF MD, the slopes were generally close to zero and did not reproduce the directional pattern observed with HFA MD stratification, and none remained statistically significant after correction. This discrepancy suggests that the apparent severity-dependent pattern observed with HFA MD stratification should be interpreted cautiously, because it may reflect HFA-based severity classification, reference-standard variability, or case-mix differences in addition to model calibration.

Interpretation of the low-SSI results requires consideration of both the reference standard and the intended use of the model. Lower SSI was associated with worse HFA sensitivity and MD in our dataset, indicating that low-SSI cases were not merely lower-quality OCT inputs but also a clinically more challenging subgroup. In such cases, higher absolute error may reflect degradation of the OCT input, greater uncertainty in the perimetric reference, or both, because lower VF sensitivity is associated with greater test–retest variability,^24^^–^^26^ and media opacity can depress global VF indices.^27^ Thus, the present analysis cannot fully separate the effect of OCT image quality from variability in the HFA reference. At the same time, this limitation is clinically relevant: The advantage of the all-SSI-trained model was observed in the subgroup in which both the OCT input and the HFA reference are likely to be most variable, which is precisely the setting encountered in routine care. For models intended for routine-care deployment, restricting training to high-SSI scans may narrow the training distribution and reduce exposure to lower-quality images encountered in clinical practice; inclusive training may therefore improve robustness to lower-SSI inputs. By contrast, when the purpose is to compare model architectures, preprocessing strategies, or augmentation methods under cleaner and more controlled conditions, restricting training and evaluation to higher-SSI scans may remain a reasonable design choice. Accordingly, our findings argue against using SSI = 7 as a rigid training exclusion threshold for routine-care model development, rather than implying that low-SSI scans are equivalent to high-SSI scans in all research contexts.

Several limitations should be acknowledged. First, this was a retrospective study using a single EfficientNet 3D-based modeling framework, and the findings may not generalize to other architectures or input representations; in particular, models based on segmentation-derived thickness maps may respond differently to low-quality scans. Second, although the dataset was multicenter, model evaluation was performed using internal patient-wise cross-validation on pooled multicenter data, and external validation or leave-one-site-out validation was not performed. Therefore, robustness to unseen institutions or different acquisition environments remains to be established. Third, all OCT data were acquired with NIDEK devices, and image-quality metrics are manufacturer specific, so extrapolation to other platforms requires direct validation. Fourth, although the supplementary size-matched analysis suggested that the advantage in low-SSI data was not explained solely by larger overall sample size, the present study was not designed to fully isolate the mechanism underlying this effect. Because matching the total number of training samples required reducing the number of higher-SSI training samples, the specific contribution of low-SSI exposure cannot be fully separated from the effect of altered training-set composition. Fifth, diagnostic labels were not uniformly available across the five participating institutions: Four sites lacked diagnosis labels for this retrospective analysis, and the remaining site had incomplete labels limited to a subset of glaucoma follow-up cases. Therefore, we could not generate a reliable multicenter case-mix table or perform a representative glaucoma-only sensitivity analysis. The diagnosis-unrestricted cohort may have included eyes with nonglaucomatous optic neuropathy, macular disease, media opacity, or other conditions, and disease-specific differences in OCT–VF coupling could not be evaluated. Finally, SSI was used as a practical summary index of image quality, but other factors, including motion artifacts, decentration, and residual OCT-HFA temporal mismatch, may also influence performance.

## Conclusions

In this multicenter retrospective study, training a segmentation-free EfficientNet 3D-based 3DCNN using OCT scans across the full range of SSIs was associated with improved estimation accuracy under low-SSI conditions, without a significant performance penalty in higher-SSI test data. Estimation error increased gradually as SSI decreased, with no clear threshold effect, indicating that image quality was better represented as a continuous rather than binary factor in this dataset. These findings suggest that, for this segmentation-free 3D OCT-based VF estimation framework, rigid SSI-based exclusion during training may reduce robustness to lower-quality scans encountered in routine-care deployment.

## Supplementary Material

Supplement 1
