# Supplementary material for: Effect of Signal-Strength Filtering on 3D Convolutional Neural Network–Based Visual Field Estimation From Macular OCT
Source: Transl Vis Sci Technol. 2026 Jul 13;15(7):17. doi: 10.1167/tvst.15.7.17 (PMC13374834; doi:10.1167/tvst.15.7.17)
Supplement: Supplement 1 [file tvst-15-7-17_s001.pdf]

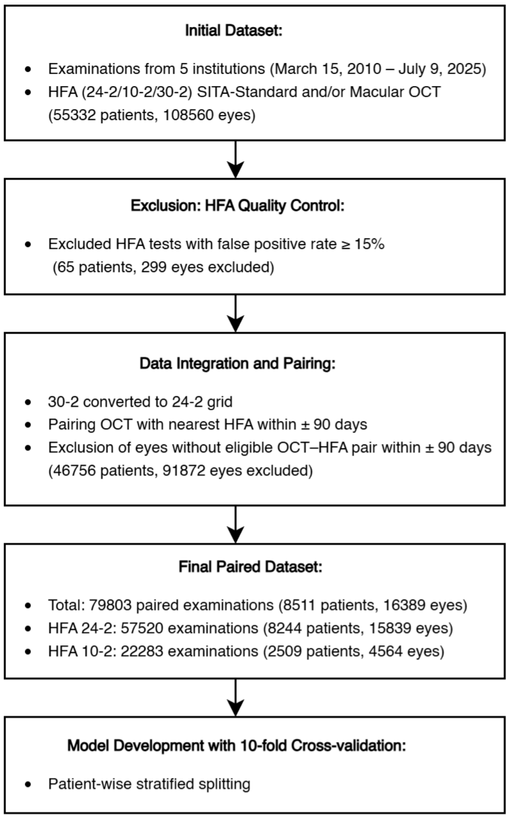

Supplementary Figure S1. Flowchart of dataset selection and pairing. Initial data were collected from five institutions. After applying the HFA reliability criterion (false-positive rate < 15%), OCT scans were paired with the temporally closest HFA examination within ± 90 days. The final dataset comprised 79,803 paired OCT and HFA examinations from 8,511 patients (16,389 eyes). No SSI-based exclusion was applied during dataset construction.

HFA, Humphrey Field Analyzer; OCT, optical coherence tomography; SSI, signal strength index.

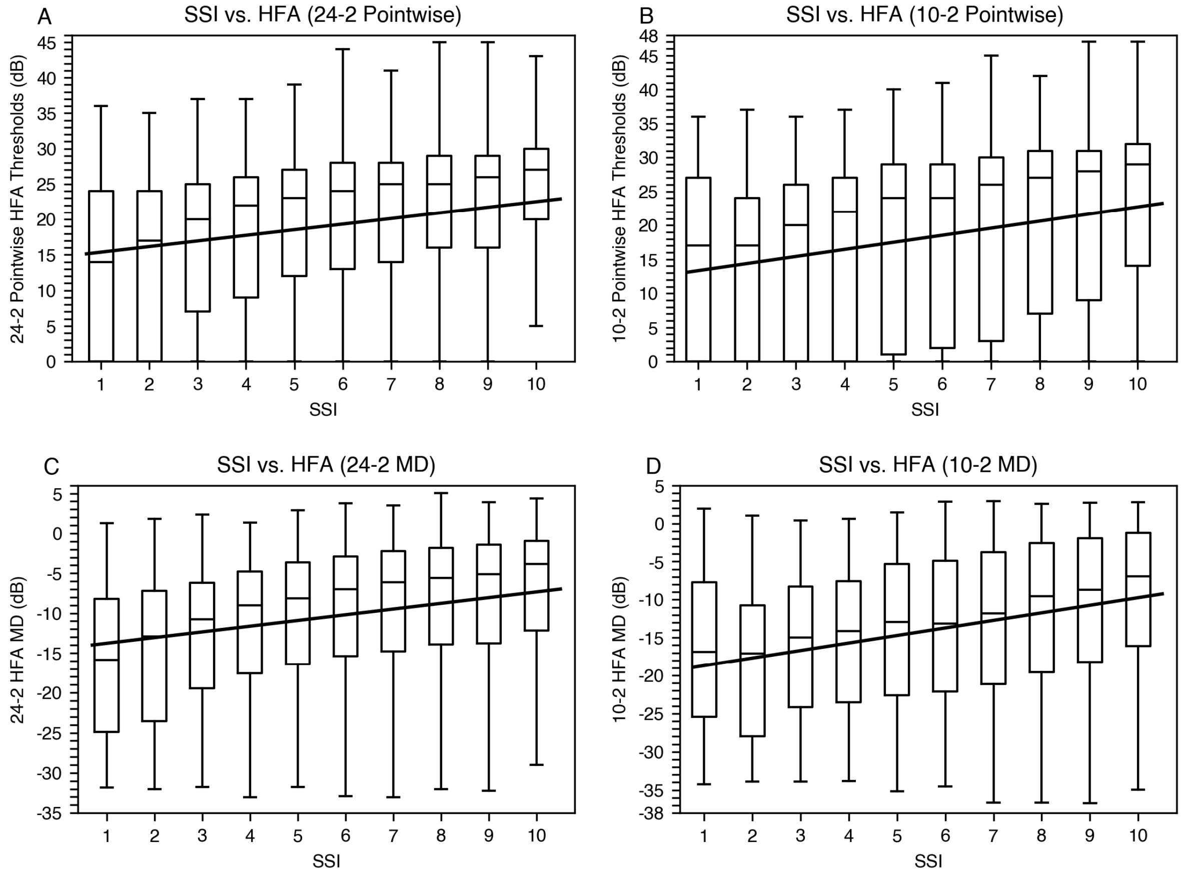

Supplementary Figure S2. Relationship between OCT SSI and HFA measurements. Boxplots show distributions of HFA values stratified by SSI (1–10). Panels show 24-2 pointwise sensitivity (A), 10-2 pointwise sensitivity (B), 24-2 MD (C), and 10-2 MD (D). Solid lines indicate linear regression fits. HFA sensitivity and MD were lower at lower SSI values. Regression slopes were 0.79, 1.04, 0.72, and 1.00 dB per unit increase in SSI for panels A through D, respectively.

OCT, optical coherence tomography; SSI, signal strength index; HFA, Humphrey Field Analyzer; MD, mean deviation.

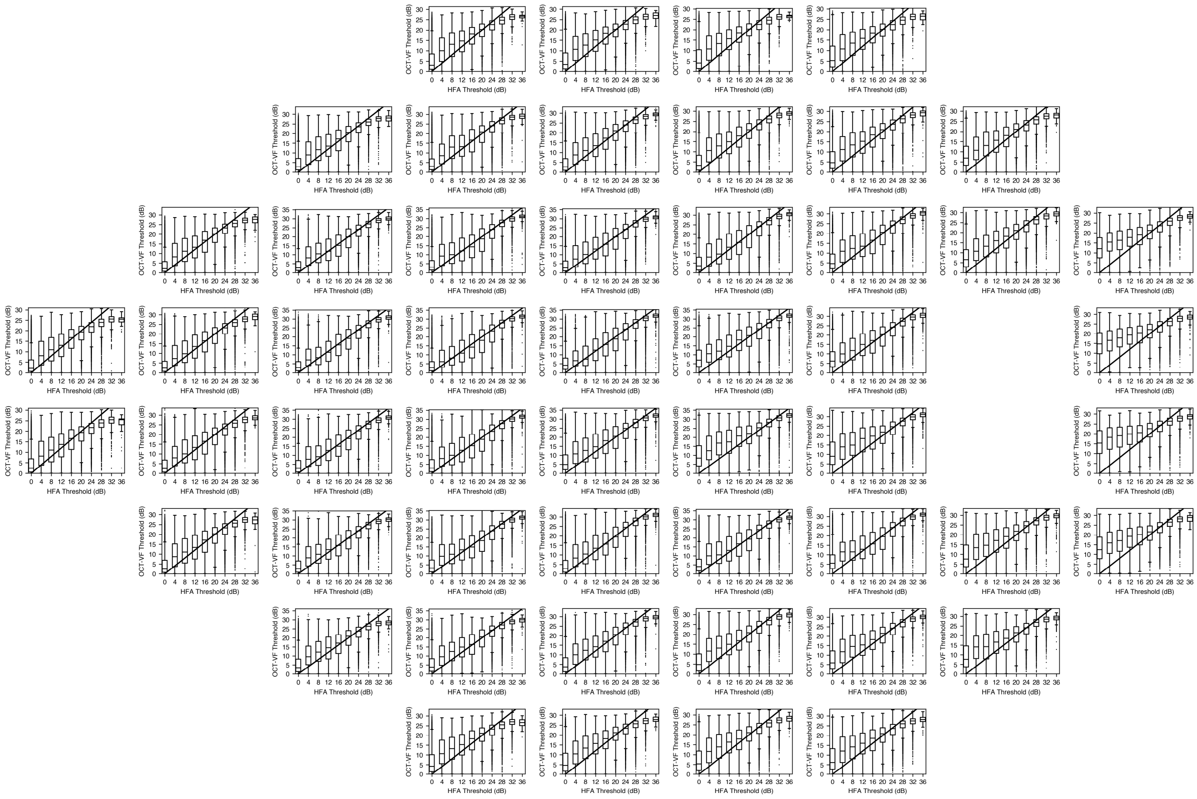

Supplementary Figure S3. Relationship between HFA measurements and OCT-based estimated visual fields (OCT-VF) for 24-2 pointwise sensitivity. Boxplots show OCT-VF values for each corresponding HFA value at all 52 test locations in the 24-2 grid. Each panel represents one test location. Left-eye data were horizontally flipped to match right-eye orientation. The diagonal line represents the line of identity (y = x). OCT-VF generally tracked HFA measurements across the dynamic range, with greater variability at lower sensitivity values. In advanced loss, deviations from HFA tended to be smaller at locations farther from the optic disc (nasal visual field) and larger at locations closer to or beyond the optic disc.

HFA, Humphrey Field Analyzer; OCT, optical coherence tomography; OCT-VF, OCT-based estimated visual field.

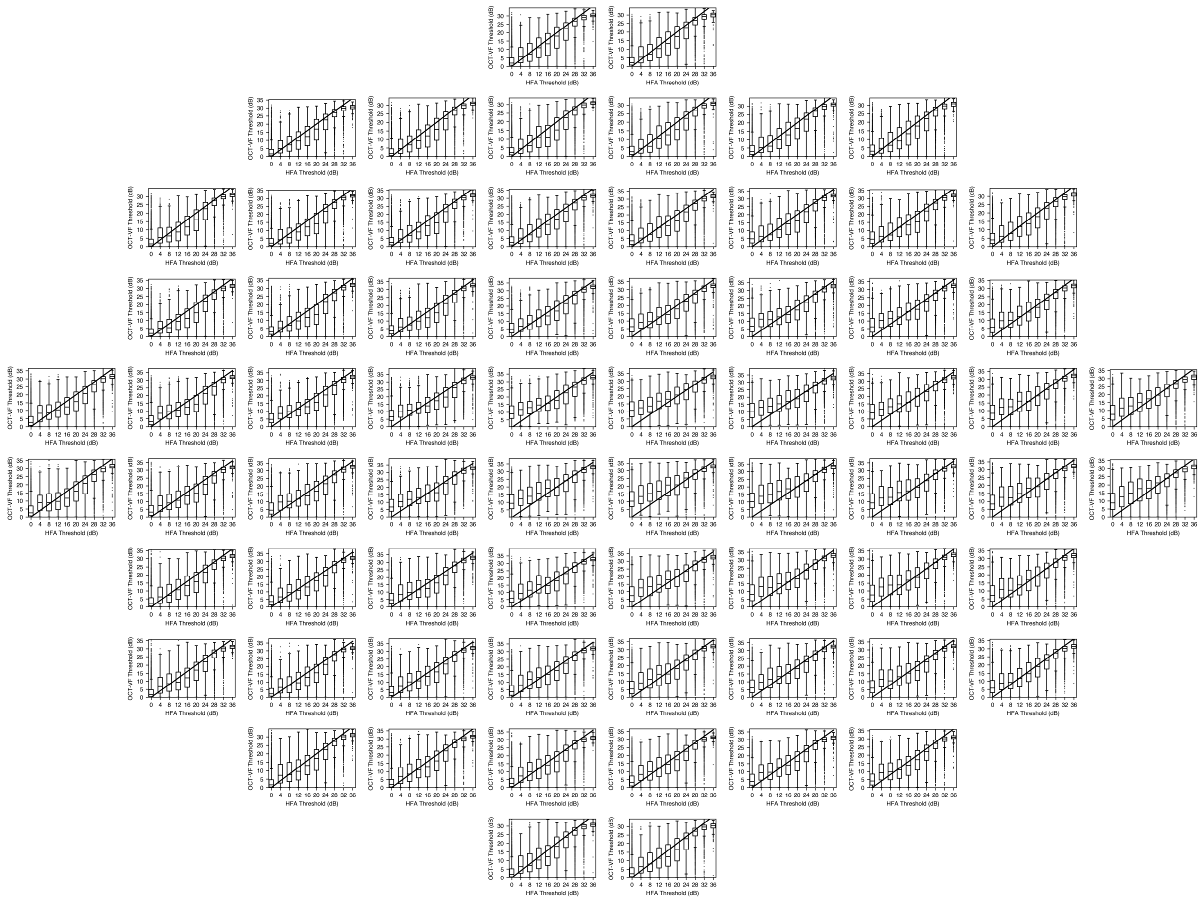

Supplementary Figure S4. Relationship between HFA measurements and OCT-based estimated visual fields (OCT-VF) for 10-2 pointwise sensitivity. Boxplots show OCT-VF values for each corresponding HFA value at all 68 test locations in the 10-2 grid. Each panel represents one test location. Left-eye data were horizontally flipped to match right-eye orientation. The diagonal line represents the line of identity (y = x). OCT-VF generally tracked HFA measurements across the dynamic range, with greater variability at lower sensitivity values. A spatial trend similar to that in the 24-2 grid was observed, particularly at lower sensitivity levels, with smaller deviations from HFA at locations farther from the optic disc and larger deviations at locations closer to the optic disc.

HFA, Humphrey Field Analyzer; OCT, optical coherence tomography; OCT-VF, OCT-based estimated visual field.

Supplementary Table S1. Comparison of Model Performance between the All-SSI-trained Model and the SSI ≥ 7-trained Model using Patient-level Bootstrap Resampling

SSI subgroup	Outcome	*n* of test samples	*n* of patients	All-SSI training	SSI ≥ 7 training	Difference [95% CI]	*P* (adjusted)
SSI ≥ 7	24-2 pointwise MAE	48732	7771	3.888	3.889	−0.002 [−0.018, 0.015]	1
SSI ≥ 7	24-2 MD absolute error	48732	7771	2.530	2.539	−0.010 [−0.034, 0.015]	1
SSI < 7	24-2 pointwise MAE	8788	3506	4.546	4.986	−0.439 [−0.502, −0.379]	< 0.001
SSI < 7	24-2 MD absolute error	8788	3506	3.118	3.535	−0.417 [−0.493, −0.342]	< 0.001
SSI ≥ 7	10-2 pointwise MAE	19523	2390	4.095	4.071	0.024 [−0.005, 0.053]	0.843
SSI ≥ 7	10-2 MD absolute error	19523	2390	2.478	2.458	0.020 [−0.028, 0.068]	1
SSI < 7	10-2 pointwise MAE	2760	1037	5.129	5.602	−0.473 [−0.594, −0.356]	< 0.001
SSI < 7	10-2 MD absolute error	2760	1037	3.420	3.873	−0.453 [−0.606, −0.305]	< 0.001

Performance was compared within SSI subgroups (SSI ≥ 7 and SSI < 7) for four prespecified endpoints: 24-2 pointwise MAE, 24-2 MD absolute error, 10-2 pointwise MAE, and 10-2 MD absolute error. Error differences were defined as the error of the all-SSI-trained model minus that of the SSI ≥ 7-trained model; negative values indicate better performance of the all-SSI-trained model. Patient-level cluster bootstrap resampling (10,000 iterations) was used to account for within-patient correlation. Bonferroni correction was applied across the eight prespecified comparisons.

SSI, signal strength index; MAE, mean absolute error; MD, mean deviation; CI, confidence interval.

Supplementary Table S2. Sensitivity Analysis Restricted to OCT-HFA Pairs Acquired within ±30 Days

**SSI subgroup**	**Outcome**	***n* of test samples**	***n* of patients**	**All-SSI training**	**SSI ≥ 7 training**	**Difference [95% CI]**	***P* (adjusted)**
SSI ≥ 7	24-2 pointwise MAE	35241	6810	3.823	3.822	0.001 [−0.017, 0.019]	1
SSI ≥ 7	24-2 MD absolute error	35241	6810	2.482	2.489	−0.007 [−0.034, 0.020]	1
SSI < 7	24-2 pointwise MAE	5925	2810	4.42	4.827	−0.406 [−0.476, −0.338]	< 0.001
SSI < 7	24-2 MD absolute error	5925	2810	3.032	3.408	−0.376 [−0.465, −0.288]	< 0.001
SSI ≥ 7	10-2 pointwise MAE	14981	2203	4.041	4.015	0.026 [−0.004, 0.056]	0.725
SSI ≥ 7	10-2 MD absolute error	14981	2203	2.446	2.420	0.027 [−0.024, 0.076]	1
SSI < 7	10-2 pointwise MAE	1858	825	4.851	5.345	−0.495 [−0.637, −0.356]	< 0.001
SSI < 7	10-2 MD absolute error	1858	825	3.254	3.706	−0.452 [−0.635, −0.270]	< 0.001

Performance was compared after restricting the held-out test samples to OCT-HFA pairs acquired within ±30 days. The same trained models, SSI subgroups, endpoints, and patient-level cluster bootstrap framework used in the primary analysis were applied. Error differences were defined as the error of the all-SSI-trained model minus that of the SSI ≥ 7-trained model; negative values indicate lower error for the all-SSI-trained model. Bonferroni correction was applied across the eight comparisons shown in this table. Values are shown in dB.

SSI, signal strength index; OCT, optical coherence tomography; HFA, Humphrey Field Analyzer; MAE, mean absolute error; MD, mean deviation; CI, confidence interval.

Supplementary Table S3. Sensitivity Analysis Restricted to Visual Fields with False-Positive Responses <15%, False-Negative Responses <15%, and Fixation Losses <15%

SSI subgroup	Outcome	*n* of test samples	*n* of patients	All-SSI training	SSI ≥ 7 training	Difference [95% CI]	*P* (adjusted)
SSI ≥ 7	24-2 pointwise MAE	35173	6725	3.68	3.674	0.006 [−0.012, 0.023]	1
SSI ≥ 7	24-2 MD absolute error	35173	6725	2.345	2.348	−0.003 [−0.030, 0.023]	1
SSI < 7	24-2 pointwise MAE	6297	2784	4.370	4.785	−0.415 [−0.486, −0.348]	< 0.001
SSI < 7	24-2 MD absolute error	6297	2784	2.967	3.357	−0.390 [−0.474, −0.311]	< 0.001
SSI ≥ 7	10-2 pointwise MAE	15534	2207	3.837	3.810	0.027 [−0.003, 0.056]	0.629
SSI ≥ 7	10-2 MD absolute error	15534	2207	2.296	2.272	0.024 [−0.026, 0.072]	1
SSI < 7	10-2 pointwise MAE	2096	872	4.900	5.388	−0.488 [−0.631, −0.351]	< 0.001
SSI < 7	10-2 MD absolute error	2096	872	3.242	3.726	−0.484 [−0.659, −0.312]	< 0.001

Performance was compared after restricting the held-out test samples to visual fields with false-positive responses <15%, false-negative responses <15%, and fixation losses <15%. The same trained models, SSI subgroups, endpoints, and patient-level cluster bootstrap framework used in the primary analysis were applied. Error differences were defined as the error of the all-SSI-trained model minus that of the SSI ≥ 7-trained model; negative values indicate lower error for the all-SSI-trained model. Bonferroni correction was applied across the eight comparisons shown in this table. Values are shown in dB.

SSI, signal strength index; MAE, mean absolute error; MD, mean deviation; CI, confidence interval.

Supplementary Table S4. Sensitivity Analysis Restricted to Native 24-2 Visual Fields

**SSI subgroup**	**Outcome**	***n* of test samples**	***n* of patients**	**All-SSI training**	**SSI ≥ 7 training**	**Difference [95% CI]**	***P* (adjusted)**
SSI ≥ 7	24-2 pointwise MAE	5,890	1,781	3.936	3.932	0.004 [−0.033, 0.040]	1
SSI ≥ 7	24-2 MD absolute error	5,890	1,781	2.590	2.592	−0.002 [−0.054, 0.049]	1
SSI < 7	24-2 pointwise MAE	1,024	557	4.430	4.842	−0.412 [−0.549, −0.278]	< 0.001
SSI < 7	24-2 MD absolute error	1,024	557	2.917	3.339	−0.422 [−0.609, −0.232]	< 0.001

Performance was compared after restricting the 24-2 held-out test samples to native 24-2 visual fields, excluding 30-2 examinations that had been truncated to the 24-2 grid. The same trained models, SSI subgroups, 24-2 endpoints, and patient-level cluster bootstrap framework used in the primary analysis were applied. Error differences were defined as the error of the all-SSI-trained model minus that of the SSI ≥ 7-trained model; negative values indicate lower error for the all-SSI-trained model. Bonferroni correction was applied across the four comparisons shown in this table. Values are shown in dB.

SSI, signal strength index; MAE, mean absolute error; MD, mean deviation; CI, confidence interval.

Supplementary Table S5. Device-Family-Specific Model Comparison in the SSI < 7 Test Subgroup

Device family	Outcome	*n* of test samples	*n* of patients	All-SSI training	SSI ≥ 7 training	Difference [95% CI]	*P* (adjusted)
RS-3000 series	24-2 pointwise MAE	8690	3461	4.536	4.970	−0.434 [−0.495, −0.371]	< 0.001
RS-3000 series	24-2 MD absolute error	8690	3461	3.110	3.518	−0.408 [−0.484, −0.330]	< 0.001
RS-3000 series	10-2 pointwise MAE	2741	1024	5.117	5.588	−0.471 [−0.590, −0.353]	< 0.001
RS-3000 series	10-2 MD absolute error	2741	1024	3.412	3.859	−0.448 [−0.601, −0.300]	< 0.001
Mirante	24-2 pointwise MAE	98	65	5.449	6.401	−0.952 [−1.630, −0.376]	0.001
Mirante	24-2 MD absolute error	98	65	3.804	5.026	−1.222 [−2.036, −0.496]	0.001
Mirante	10-2 pointwise MAE	19	14	6.906	7.685	−0.779 [−1.922, 0.335]	0.361
Mirante	10-2 MD absolute error	19	14	4.575	5.768	−1.193 [−2.931, 0.676]	0.361

Performance was compared within the SSI < 7 held-out test subgroup after stratification by Nidek device family. RS-3000, RS-3000 Advance 2, and RS-330 were grouped as the RS-3000 series, and Mirante was analyzed separately. Error differences were defined as the error of the all-SSI-trained model minus that of the SSI ≥ 7-trained model; negative values indicate lower error for the all-SSI-trained model. Patient-level cluster bootstrap resampling was used. Holm correction was applied across the eight comparisons shown in this table. Values are shown in dB. This analysis was intended to assess whether the direction of the low-SSI performance difference was consistent across device families. Estimates for Mirante, particularly for 10-2 outcomes, should be interpreted cautiously because of the small sample size.

SSI, signal strength index; MAE, mean absolute error; MD, mean deviation; CI, confidence interval.

Supplementary Table S6. Device-by-SSI Interaction Analysis of Between-Model Differences

**Outcome**	**Total *n* of test samples**	**Total *n* of patients**	**Interaction estimate [95% CI]**	***P* (adjusted)**
24-2 pointwise MAE	57520	8244	−0.533 [−1.171, 0.105]	0.305
24-2 MD absolute error	57520	8244	−0.765 [−1.546, 0.015]	0.219
10-2 pointwise MAE	22283	2509	−0.197 [−1.305, 0.911]	1
10-2 MD absolute error	22283	2509	−0.576 [−2.363, 1.211]	1

The paired error difference was defined as the error of the all-SSI-trained model minus that of the SSI ≥ 7-trained model. Linear models were fitted with the paired error difference as the dependent variable and SSI subgroup, device family, and their interaction as explanatory variables. RS-3000, RS-3000 Advance 2, and RS-330 were grouped as the RS-3000 series, and Mirante was analyzed as the other device family. The interaction estimate represents the difference in the SSI-dependent between-model error difference for Mirante relative to the RS-3000 series. Negative values indicate that the additional advantage of the all-SSI-trained model in SSI < 7 data was larger for Mirante than for the RS-3000 series. Cluster-robust standard errors were computed with patient ID as the clustering variable. Holm correction was applied across the four endpoint-specific interaction tests. The total n rows and total n patients indicate the number of paired observations and patients included in each interaction model, including both RS-3000 series and Mirante devices. Device-family-specific sample sizes are shown in Supplementary Table S5.
SSI, signal strength index; MAE, mean absolute error; MD, mean deviation; CI, confidence interval.

Supplementary Table S7. Fold-wise Comparison of Model Performance using the Wilcoxon Signed-rank Test

SSI subgroup	Outcome	All-SSI training	SSI ≥ 7 training	Difference	*P* (Wilcoxon)	*P* (adjusted)
SSI ≥ 7	24-2 pointwise MAE	3.887	3.888	−0.001	0.695	1
SSI ≥ 7	24-2 MD absolute error	2.530	2.538	−0.009	1	1
SSI < 7	24-2 pointwise MAE	4.549	4.990	−0.442	0.002	0.0156
SSI < 7	24-2 MD absolute error	3.122	3.540	−0.418	0.002	0.0156
SSI ≥ 7	10-2 pointwise MAE	4.092	4.068	0.024	0.322	1
SSI ≥ 7	10-2 MD absolute error	2.477	2.456	0.021	0.492	1
SSI < 7	10-2 pointwise MAE	5.116	5.592	−0.476	0.002	0.0156
SSI < 7	10-2 MD absolute error	3.403	3.861	−0.457	0.002	0.0156

For each of the 10 cross-validation folds, mean errors were calculated separately for the all-SSI-trained model and the SSI ≥ 7-trained model. Paired between-model comparisons were then performed with the Wilcoxon signed-rank test. Results are shown for each SSI subgroup and endpoint. Bonferroni correction was applied across the eight comparisons.

SSI, signal strength index; MAE, mean absolute error; MD, mean deviation.

Supplementary Table S8. Additive Low-SSI Exposure Analysis in the SSI < 7 Test Subgroup

Training strategy	24-2 pointwise MAE	24-2 MD absolute error	10-2 pointwise MAE	10-2 MD absolute error
SSI ≥ 7 training	4.986	3.535	5.602	3.873
SSI ≥ 7 + 25% low-SSI training	4.637	3.224	5.187	3.466
SSI ≥ 7 + 50% low-SSI training	4.575	3.187	5.165	3.473
All-SSI training	4.546	3.118	5.129	3.420

Mean errors are shown for models trained with progressively increasing exposure to low-SSI scans while retaining all high-SSI training scans. Low-SSI scans were defined as scans with SSI < 7, and high-SSI scans were defined as scans with SSI ≥ 7. The SSI ≥ 7 training model used only high-SSI scans for training. The 25% and 50% low-SSI models retained all high-SSI training scans and additionally included 25% or 50% of the available low-SSI training scans, respectively. Low-SSI scans were subsampled separately within the 24-2 and 10-2 training datasets while preserving the SSI distribution as closely as possible. The All-SSI model used all available training scans regardless of SSI. Performance was evaluated only in the SSI < 7 held-out test subgroup. The values for SSI ≥ 7 training and All-SSI training are reproduced from Supplementary Table S1 to provide reference endpoints for the additive low-SSI exposure analysis. Values represent mean pointwise MAE or mean absolute MD error in dB. Lower values indicate better performance. SSI, signal strength index; MAE, mean absolute error; MD, mean deviation.

Supplementary Table S9. Comparison of Model Performance between the Size-matched All-SSI-trained Model and the SSI ≥ 7-trained Model using Patient-level Bootstrap Resampling

SSI subgroup	Outcome	*n* of test samples	*n* of patients	Size-matched all-SSI training	SSI ≥ 7 training	Difference [95% CI]	*P* (adjusted)
SSI ≥ 7	24-2 pointwise MAE	48732	7771	3.948	3.889	0.059 [0.042, 0.075]	< 0.001
SSI ≥ 7	24-2 MD absolute error	48732	7771	2.593	2.539	0.054 [0.029, 0.078]	< 0.001
SSI < 7	24-2 pointwise MAE	8788	3506	4.596	4.986	−0.390 [−0.451, −0.330]	< 0.001
SSI < 7	24-2 MD absolute error	8788	3506	3.162	3.535	−0.372 [−0.450, −0.297]	< 0.001
SSI ≥ 7	10-2 pointwise MAE	19523	2390	4.151	4.071	0.080 [0.048, 0.113]	< 0.001
SSI ≥ 7	10-2 MD absolute error	19523	2390	2.550	2.458	0.092 [0.038, 0.148]	0.002
SSI < 7	10-2 pointwise MAE	2760	1037	5.121	5.602	−0.482 [−0.623, −0.352]	< 0.001
SSI < 7	10-2 MD absolute error	2760	1037	3.418	3.873	−0.454 [−0.626, −0.295]	< 0.001

Performance was compared within SSI subgroups (SSI ≥ 7 and SSI < 7) for four prespecified endpoints: 24-2 pointwise MAE, 24-2 MD absolute error, 10-2 pointwise MAE, and 10-2 MD absolute error. The size-matched all-SSI-trained model was created by downsampling the all-SSI training set to exactly match the number of training samples in the SSI ≥ 7-trained model while preserving the original MD distribution as closely as possible by systematic subsampling after sorting by MD. Error differences were defined as the error of the size-matched all-SSI-trained model minus that of the SSI ≥ 7-trained model; negative values indicate better performance of the size-matched all-SSI-trained model. Patient-level cluster bootstrap resampling (10,000 iterations) was used to account for within-patient correlation. Bonferroni correction was applied across the eight prespecified comparisons.

SSI, signal strength index; MAE, mean absolute error; MD, mean deviation; CI, confidence interval.

Supplementary Table S10. MD-stratified Model Comparison in the SSI < 7 Test Subgroup

Severity stratum	Outcome	*n* of test samples	*n* of patients	All-SSI training	SSI ≥ 7 training	Difference [95% CI]	*P* (adjusted)
Mild (MD > −6 dB)	24-2 pointwise MAE	3503	1870	3.023	3.308	−0.285 [−0.362, −0.208]	< 0.001
Mild (MD > −6 dB)	24-2 MD absolute error	3503	1870	2.280	2.518	−0.238 [−0.324, −0.153]	< 0.001
Mild (MD > −6 dB)	10-2 pointwise MAE	735	360	3.118	3.34	−0.222 [−0.416, −0.036]	0.035
Mild (MD > −6 dB)	10-2 MD absolute error	735	360	2.606	2.803	−0.197 [−0.414, 0.016]	0.068
Moderate (−12 dB < MD ≤ −6 dB)	24-2 pointwise MAE	1995	1096	4.768	5.432	−0.664 [−0.788, −0.539]	< 0.001
Moderate (−12 dB < MD ≤ −6 dB)	24-2 MD absolute error	1995	1096	2.980	3.762	−0.782 [−0.942, −0.618]	< 0.001
Moderate (−12 dB < MD ≤ −6 dB)	10-2 pointwise MAE	508	268	4.823	5.629	−0.806 [−1.138, −0.474]	< 0.001
Moderate (−12 dB < MD ≤ −6 dB)	10-2 MD absolute error	508	268	3.308	4.212	−0.904 [−1.273, −0.539]	< 0.001
Severe (MD ≤ −12 dB)	24-2 pointwise MAE	3290	1390	6.034	6.502	−0.467 [−0.574, −0.365]	< 0.001
Severe (MD ≤ −12 dB)	24-2 MD absolute error	3290	1390	4.093	4.479	−0.386 [−0.523, −0.251]	< 0.001
Severe (MD ≤ −12 dB)	10-2 pointwise MAE	1517	635	6.206	6.689	−0.483 [−0.642, −0.326]	< 0.001
Severe (MD ≤ −12 dB)	10-2 MD absolute error	1517	635	3.852	4.277	−0.425 [−0.640, −0.213]	< 0.001

The SSI < 7 held-out test subgroup was stratified by HFA mean deviation (MD) into mild (MD > −6 dB), moderate (−12 dB < MD ≤ −6 dB), and severe (MD ≤ −12 dB) strata. Within each severity stratum, model performance was compared between the all-SSI-trained model and the SSI ≥ 7-trained model using patient-level cluster bootstrap resampling. Differences were defined as the error of the all-SSI-trained model minus

that of the SSI ≥ 7-trained model; negative values indicate lower error for the all-SSI-trained model. P values were calculated by two-sided patient-level cluster bootstrap resampling. Adjusted P values were Bonferroni-corrected across the 12 comparisons shown in this table. Values are shown in dB.
SSI, signal strength index; HFA, Humphrey Field Analyzer; MD, mean deviation; MAE, mean absolute error; CI, confidence interval.

Supplementary Table S11. Interaction Analysis of Between-Model Differences According to SSI Subgroup

Outcome	Contrast	Estimate [95% CI]	*P* (raw)	*P* (adjusted)
24-2 Pointwise MAE	Difference at SSI ≥ 7	−0.0016 [−0.0185 to 0.0152]	0.849	1
24-2 Pointwise MAE	Additional change at SSI < 7	−0.4376 [−0.4981 to −0.3772]	< 0.001	< 0.001
24-2 Pointwise MAE	Difference at SSI < 7	−0.4393 [−0.4999 to −0.3786]	< 0.001	< 0.001
24-2 MD absolute error	Difference at SSI ≥ 7	−0.0095 [−0.0346 to 0.0155]	0.455	1
24-2 MD absolute error	Additional change at SSI < 7	−0.4075 [−0.4820 to −0.3329]	< 0.001	< 0.001
24-2 MD absolute error	Difference at SSI < 7	−0.4170 [−0.4921 to −0.3419]	< 0.001	< 0.001
10-2 Pointwise MAE	Difference at SSI ≥ 7	0.02370 [−0.0052 to 0.0527]	0.108	1
10-2 Pointwise MAE	Additional change at SSI < 7	−0.4968 [−0.6157 to −0.3779]	< 0.001	< 0.001
10-2 Pointwise MAE	Difference at SSI < 7	−0.4731 [−0.5932 to −0.3530]	< 0.001	< 0.001
10-2 MD absolute error	Difference at SSI ≥ 7	0.0204 [−0.0274 to 0.0682]	0.402	1
10-2 MD absolute error	Additional change at SSI < 7	−0.4731 [−0.6234 to −0.3228]	< 0.001	< 0.001
10-2 MD absolute error	Difference at SSI < 7	−0.4527 [−0.6035 to −0.3019]	< 0.001	< 0.001

The table presents results from a linear model evaluating between-model differences in estimation error (all-SSI-trained model minus SSI ≥ 7-trained model) and their modification by SSI subgroup.
For each outcome, the intercept represents the between-model difference in the SSI ≥ 7 subgroup, and the coefficient for SSI < 7 represents the additional change in the SSI < 7 subgroup (interaction term). The between-model difference in the SSI < 7 subgroup corresponds to the sum of these two terms. Negative values indicate better performance of the all-SSI-trained model.
P (raw) indicates the unadjusted *P* value for each reported term or contrast. *P* (adjusted) indicates Bonferroni-adjusted *P* values across the 12 prespecified tests.
Estimates are presented with 95% confidence intervals based on cluster-robust standard errors clustered by patient ID.
SSI, signal strength index; MAE, mean absolute error; CI, confidence interval; MD, mean deviation.

Supplementary Table S12. Association between SSI and Signed Error Estimated by Patient-level Cluster Bootstrap

Outcome	*n* of test samples	*n* of patients	Slope per SSI [95% CI]
24-2 Pointwise signed error	57520	8244	–0.036 [–0.074 to 0.001]
24-2 MD signed error	57520	8244	–0.013 [–0.052 to 0.024]
10-2 Pointwise signed error	22283	2509	–0.06 [–0.131 to 0.012]
10-2 MD signed error	22283	2509	–0.033 [–0.107 to 0.043]

Linear regression models were fitted with signed error (OCT-based estimate minus HFA measurement) as the dependent variable and SSI as the explanatory variable. Patient-level cluster bootstrap resampling (10,000 iterations) was performed to account for within-patient correlation. The slope represents the change in signed error per one-unit increase in SSI. Estimates are presented with 95% confidence intervals derived from the bootstrap distribution. The corresponding signed-error plots are shown in Figure 5. OCT-based estimates were generated using the all-SSI-trained model and evaluated on the full dataset.

SSI, signal strength index; OCT, optical coherence tomography; HFA, Humphrey Field Analyzer; CI, confidence interval; MD, mean deviation.

Supplementary Table S13. Association between SSI and Signed Error Stratified by HFA MD Severity

**Severity stratum**	**Outcome**	***n* of test samples**	***n* of patients**	**Slope per SSI [95% CI]**	***P* (adjusted)**
Mild (MD > −6 dB)	24-2 pointwise signed error	29433	6105	0.188 [0.155, 0.220]	< 0.001
Mild (MD > −6 dB)	24-2 MD signed error	29433	6105	0.253 [0.220, 0.285]	< 0.001
Mild (MD > −6 dB)	10-2 pointwise signed error	8793	1473	0.248 [0.168, 0.327]	< 0.001
Mild (MD > −6 dB)	10-2 MD signed error	8793	1473	0.326 [0.244, 0.407]	< 0.001
Moderate (−12 dB < MD ≤ −6 dB)	24-2 pointwise signed error	10636	2937	0.119 [0.054, 0.188]	0.007
Moderate (−12 dB < MD ≤ −6 dB)	24-2 MD signed error	10636	2937	0.123 [0.056, 0.193]	0.010
Moderate (−12 dB < MD ≤ −6 dB)	10-2 pointwise signed error	3858	878	0.026 [−0.118, 0.176]	1
Moderate (−12 dB < MD ≤ −6 dB)	10-2 MD signed error	3858	878	0.033 [−0.107, 0.180]	1
Severe (MD ≤ −12 dB)	24-2 pointwise signed error	17451	2966	−0.115 [−0.181, −0.046]	0.014
Severe (MD ≤ −12 dB)	24-2 MD signed error	17451	2966	−0.131 [−0.201, −0.058]	0.007
Severe (MD ≤ −12 dB)	10-2 pointwise signed error	9632	1447	−0.125 [−0.232, −0.024]	0.202
Severe (MD ≤ −12 dB)	10-2 MD signed error	9632	1447	−0.134 [−0.249, −0.023]	0.223

Linear regression models were fitted with signed error, defined as the OCT-based estimate minus the HFA measurement, as the dependent variable and SSI as the explanatory variable. Analyses were stratified by HFA mean deviation (MD) severity: mild (MD > −6 dB), moderate (−12 dB < MD ≤ −6 dB), and severe (MD ≤ −12 dB). Patient-level cluster bootstrap resampling with 10,000 iterations was used to account for within-patient correlation. Slopes represent the change in signed error per one-unit increase in displayed SSI. Positive slopes indicate that signed error became more negative at lower SSI values, whereas negative slopes indicate that signed error became more positive at lower SSI values. Adjusted P values were Bonferroni-corrected across the 12 comparisons shown in this table. OCT-based estimates were generated using the all-SSI-trained model.

SSI, signal strength index; HFA, Humphrey Field Analyzer; MD, mean deviation; OCT, optical coherence tomography; CI, confidence interval.

Supplementary Table S14. Association between SSI and Signed Error Stratified by OCT-VF MD Severity

Severity stratum	Outcome	*n* of test samples	*n* of patients	Slope per SSI [95% CI]	*P* (adjusted)
Mild (OCT-VF MD > −6 dB)	24-2 pointwise signed error	29886	6329	−0.038 [−0.080, 0.003]	0.838
Mild (OCT-VF MD > −6 dB)	24-2 MD signed error	29886	6329	0.032 [−0.011, 0.074]	1
Mild (OCT-VF MD > −6 dB)	10-2 pointwise signed error	8565	1454	−0.118 [−0.225, −0.018]	0.214
Mild (OCT-VF MD > −6 dB)	10-2 MD signed error	8565	1454	−0.040 [−0.156, 0.069]	1
Moderate (−12 dB < OCT-VF MD ≤ −6 dB)	24-2 pointwise signed error	10972	3016	−0.087 [−0.164, −0.011]	0.31
Moderate (−12 dB < OCT-VF MD ≤ −6 dB)	24-2 MD signed error	10972	3016	−0.081 [−0.161, −0.004]	0.48
Moderate (−12 dB < OCT-VF MD ≤ −6 dB)	10-2 pointwise signed error	3833	895	−0.054 [−0.189, 0.088]	1
Moderate (−12 dB < OCT-VF MD ≤ −6 dB)	10-2 MD signed error	3833	895	−0.056 [−0.193, 0.089]	1
Severe (OCT-VF MD ≤ −12 dB)	24-2 pointwise signed error	16662	2682	0.000 [−0.067, 0.067]	1
Severe (OCT-VF MD ≤ −12 dB)	24-2 MD signed error	16662	2682	−0.027 [−0.097, 0.043]	1
Severe (OCT-VF MD ≤ −12 dB)	10-2 pointwise signed error	9885	1442	−0.031 [−0.136, 0.070]	1
Severe (OCT-VF MD ≤ −12 dB)	10-2 MD signed error	9885	1442	−0.041 [−0.153, 0.066]	1

Linear regression models were fitted with signed error, defined as the OCT-based VF estimate minus the HFA measurement, as the dependent variable and SSI as the explanatory variable. Analyses were stratified by OCT-VF MD severity: mild (OCT-VF MD > −6 dB), moderate (−12 dB < OCT-VF MD ≤ −6 dB), and severe (OCT-VF MD ≤ −12 dB). Patient-level cluster bootstrap resampling with 10,000 iterations was used to account for within-patient correlation. Slopes represent the change in signed error per one-unit increase in displayed SSI. Adjusted P values were Bonferroni-corrected across the 12 comparisons shown in this table. This analysis was performed as an exploratory sensitivity analysis using model-derived OCT-VF MD, rather than HFA MD, to define severity strata.

SSI, signal strength index; OCT-VF, OCT-based estimated visual field; HFA, Humphrey Field Analyzer; MD, mean deviation; CI, confidence interval.

Supplementary Table S15. Error of OCT-VF Relative to HFA

Outcome	MAE (dB)	ME (dB)
24-2 Pointwise	3.99 ± 4.37	0.15 ± 5.92
10-2 Pointwise	4.22 ± 4.71	0.00 ± 6.32
24-2 MD	2.62 ± 2.70	0.20 ± 3.75
10-2 MD	2.59 ± 2.60	–0.03 ± 3.67

Mean absolute error (MAE) and mean error (ME) were calculated between OCT-VF estimates and corresponding HFA measurements for each parameter. Mean error was defined as OCT-VF minus HFA. Values are presented as mean ± standard deviation. Estimates were generated by the all-SSI-trained model and evaluated on the full dataset.

OCT, optical coherence tomography; OCT-VF, OCT-based estimated visual field; HFA, Humphrey Field Analyzer; MAE, mean absolute error; ME, mean error; MD, mean deviation.
